# Cerebrospinal fluid matrix metalloproteinase-9 increases during treatment of recurrent malignant gliomas

**DOI:** 10.1186/1743-8454-5-1

**Published:** 2008-01-11

**Authors:** Eric T Wong, David Alsop, Diana Lee, Angela Tam, Loretta Barron, Julianne Bloom, Shiva Gautam, Julian K Wu

**Affiliations:** 1 Beth Israel Deaconess Medical Center, Brain Tumor Center & Neuro-Oncology Unit, Boston, MA 02215, USA; 2 Beth Israel Deaconess Medical Center, Department of Neurology, Boston, MA 02215, USA; 3 Beth Israel Deaconess Medical Center, Division of Neuroradiology, Boston, MA 02215, USA; 4 Beth Israel Deaconess Medical Center, Division of Biostatistics, Boston, MA 02215, USA; 5 Beth Israel Deaconess Medical Center, Division of Neurosurgery, Boston, MA 02215, USA; 6 Tufts-New England Medical Center, Department of Neurosurgery, Boston, MA 02111, USA

## Abstract

**Background:**

Matrix metalloproteinases (MMPs) are enzymes that promote tumor invasion and angiogenesis by enzymatically remodeling the extracellular matrix. MMP-2 and MMP-9 are the most abundant forms of MMPs in malignant gliomas, while a 130 kDa MMP is thought to be MMP-9 complexed to other proteinases. This study determined whether doxycycline can block MMP activity *in vitro*. We also measured MMP-2 and MMP-9 levels in cerebrospinal fluid (CSF) from patients with recurrent malignant gliomas.

**Methods:**

To determine whether doxycycline can block MMP activity, we measured the extent of doxycyline-mediated MMP-2 and MMP-9 inhibition *in vitro *using epidermal growth factor receptor (EGFR) transfected U251 glioma cell lines. MMP activity was measured using sodium dodecyl sulfate-polyacrylamide gel electrophoresis (SDS-PAGE) zymography. In addition, patients underwent lumbar puncture for CSF sampling at baseline, after 6 weeks (1 cycle), and after 12 weeks (2 cycles), while being treated with a novel chemotherapy regimen of irinotecan, thalidomide, and doxycycline designed to block growth/proliferation, angiogenesis, and invasion. Irinotecan was given at 125 mg/m^2^/week for 4 weeks in 6-week cycles, together with continuous doxycycline at 100 mg twice daily on Day 1 and 50 mg twice daily thereafter. Daily thalidomide dose in our cohort was 400 mg. Tumor progression was monitored by magnetic resonance imaging (MRI).

**Results:**

Doxycyline *in vitro *completely abolished MMP-9 activity at 500 μg/ml while there was only 30 to 50% inhibition of MMP-2 activity. Four patients respectively completed 4, 3, 1, and 2 cycles of irinotecan, thalidomide, and doxycycline. Patient enrollment was terminated after one patient developed radiologically defined pulmonary embolism, and another had probable pulmonary embolism. Although CSF MMP-2 and 130 kDa MMP levels were stable, MMP-9 level progressively increased during treatment despite stable MRI.

**Conclusion:**

Doxycycline can block MMP-2 and MMP-9 activities from glioma cells *in vitro*. Increased CSF MMP-9 activity could be a biomarker of disease activity in patients with malignant gliomas, before any changes are detectable on MRI.

## Background

Matrix metalloproteinases (MMPs) are Zn^2+^- or Ca^2+^-dependent proteinases that remodel the extracellular matrix during development and cancer metastasis [1,2]. In malignant gliomas, MMP-2 and MMP-9 are the most abundant forms of MMPs [3-5], while the 130 kDa MMP may represent a complex of MMP-9 and tissue inhibitor of matrix metalloproteinase-1, or a dimer of MMP-9 [6-8]. Both MMP-2 and MMP-9 have to be cleaved, either by an inefficient auto-activation process or by other active proteinases, in order to become activated metalloproteinases. Their activities can be blocked by sequestration of Zn^2+ ^or Ca^2+ ^[1,2]. Alternatively, tetracycline and its congeners, including doxycycline, may inhibit MMP activities [9,10].

Malignant gliomas are characterized by three major physiological processes: growth/proliferation, angiogenesis, and invasion [11]. Traditional cytotoxic chemotherapies control malignant gliomas by blocking growth and proliferation mechanisms, but leave angiogenesis and invasion unchecked. Furthermore, Macdonald's response criteria [12], an established magnetic resonance imaging (MRI) measure of cytotoxic chemotherapy efficacy, cannot adequately measure antiangiogenic and anti-invasion drug effects. For example, putative MMP inhibitors designed to block tumor cell migration, like marimastat and prinomastat, have undergone phase II clinical trials for malignant gliomas in combination with cytotoxic chemotherapies [13,14]; but there was no radiographic response and no prolongation of patient survival. It remains unclear whether such disappointing results were due to insufficient inhibition of MMPs or alternative mechanisms of tumor cell migration. Therefore, separate measures of drug efficacy for growth/proliferation, angiogenesis, and invasion may be necessary.

In this study we determined whether or not doxycycline could block MMP-2 and MMP-9 activities, by measurements of doxycyline-mediated MMP-2 and MMP-9 inhibition *in vitro *using epidermal growth factor receptor (EGFR) transfected U251 glioma cell lines. We also incorporated doxycyline in a phase I clinical trial using a novel chemotherapy regimen of irinotecan, thalidomide, and doxycycline to treat recurrent malignant gliomas. In this trial, we determined the cerebrospinal fluid (CSF) MMP activities in glioma patients, in an attempt to correlate MMPs with malignant glioma activity *in vivo*.

## Methods

### *In vitro *inhibitory activity of doxycycline against matrix metalloproteinases

In order to demonstrate the inhibitory activity of doxycycline on MMPs secreted by glioma cells, U251 human glioma cells stably transfected with a full-length EGFR in pcDNA3.1neo (Invitrogen, Carlsbad, CA, USA) were grown in Dulbecco's modified Eagle's medium (DMEM) with 10% fetal bovine serum to mid-log phase. Cells were then treated in serum-free DMEM for 72 hours. Ten μl of supernatant was treated with 0, 10, 50, 100, 500, and 1,000 μg/ml of doxycycline, and the MMP gelanolytic activities were evaluated by zymography (see CSF SDS-PAGE Zymography). The experiments were performed in triplicate.

### Patient selection

All patients signed informed consent for participation in this clinical trial, as well as the accompanied neuroimaging and CSF analyses. They were also required to participate in FDA-mandated System for Thalidomide Education and Prescribing Safety (S.T.E.P.) program. The procedures for this trial were in accordance with the ethical standards of the Committee for Clinical Investigation at Beth Israel Deaconess Medical Center and with the Helsinki Declaration of 1975, as revised in 1983.

Four patients were recruited using the following procedures: Adults were identified with recurrent malignant gliomas, including glioblastoma multiforme, anaplastic astrocytoma, anaplastic oligodendroglioma, mixed anaplastic oligoastrocytoma, and anaplastic ganglioglioma, after initial surgery and involved-field cranial irradiation. Additional inclusion criteria include age ≥ 18; Karnofsky Performance Score (KPS) ≥ 60; bi-dimensionally measurable disease on gadolinium-enhanced head MRI; and stable corticosteroid dose for at least 3 days before enrollment. Patients must have had adequate hematological profiles, including absolute neutrophils ≥ 1,500/mm^3^, white blood cells ≥ 3,000/mm^3^, and platelets ≥ 100,000/mm^3^. Serum blood urea nitrogen, creatinine, total bilirubin, and direct bilirubin must have been ≤ 1.5 × upper limit laboratory normal, and also ≤ 3.0 × upper limits of laboratory normal for alanine aminotransferase and aspartate aminotransferase and ≤ 2.0 × upper limit of laboratory normal for liver-derived alkaline phosphatase.

Patients were excluded if they had multifocal gliomas, gliomatosis cerebri, low-grade gliomas, or leptomeningeal spread of malignant gliomas. Additional exclusion criteria included a KPS < 60; chemotherapy, immunotherapy, or biologic therapy within 4 weeks prior to enrollment; inadequate recovery from prior therapies; poor medical risks; pre-existing peripheral neuropathy; P450 hepatic enzyme-inducing anticonvulsant intake (including phenytoin, carbamazepine, and phenobarbital); concurrent malignancies other than surgically cured carcinoma *in situ *of the cervix, and basal cell and squamous cell carcinoma of the skin; known HIV infection or AIDS-related illnesses; pregnant or nursing women; and inability to undergo FDA-mandatory S.T.E.P. program.

### Treatment schedule and response assessment

Patients received standard intravenous irinotecan at 125 mg/m^2^/week for 4 weeks in 6-week cycles [15]. On day 1 of irinotecan, they also took oral doxycycline at a dose of 100 mg twice daily, followed by 50 mg twice daily continuously thereafter. Daily thalidomide at a dose of 400 mg was also started on day 1 of irinotecan [16,17]. Patients received irinotecan, thalidomide, and doxycycline until disease progression or toxicity.

Tumor response was assessed by MRI every 6 weeks according to Macdonald's criteria [12]. High-resolution T1- and T2-weighted, gradient-echo, diffusion, arterial spin labeling (ASL) perfusion, and post gadolinium T1-weighted images (on axial, coronal, and sagittal axes) of the brain were acquired on a 3.0-Tesla MRI scanner (General Electric Healthcare Technologies, VH/I, Waukesha, WI, USA). A complete response was defined as resolution of all enhancements on MRI, while a partial response was ≥ 50% reduction of the product of bi-dimensional diameter of the tumor. Progressive disease was defined as ≥ 25% increase in size of the enhancing tumor, new tumor, or worsening neurological status requiring an increase in dose of corticosteroid. Everything else would be classified as stable disease. No prophylactic anticoagulation was prescribed unless there was a documented thromboembolic event.

### CSF SDS-PAGE zymography

The ethics committee approved three lumbar punctures per patient only, because these procedures were not standard of care for managing recurrent malignant gliomas. In order to obtain the most informative data, CSF samples were taken from patients before (baseline), after 6 weeks (1 cycle), and after 12 weeks (2 cycles) of treatment, for routine analysis and zymography. Samples for zymography were stored immediately at -80°F until batch analysis. The preparation of CSF zymography gels was previously described [18]. Briefly, zymography gels were made from 0.1% gelatin (Sigma, St. Louis, MO, USA) loading gels and 7.5% acrylamide (Sigma) stacking gels, and allowed to polymerize by adding 200 μl of ammonium persulfate (Sigma) and 50 μl of TEMED (Sigma). Twenty-five μl of CSF was loaded into polymerized gel for electrophoresis in a running buffer (25 mM Tris, 190 mM glycine, and 3.5 mM SDS) at 25 mA for 1 hour. After electrophoresis, gels were transferred into 2.5% tritionX wash for 1 hour, followed by an overnight incubation at 37°C with digestion buffer (10 mM calcium chloride and 20 mM tris acetate at pH of 7.5). Gels were stained for 1 hour with Coomassie brilliant blue R-250 (Bio-Rad, Hercules, CA, USA), followed by destaining in a solution containing 10% acetic acid and 5% methanol for 3 hours.

The MMP-2, MMP-9, and 130 kDa gelanolytic activities were measured by scoring the intensity of bands by computerized image analysis (Odessey^® ^Imaging System, LI-COR Biotechnology, Lincoln, NE, USA). The CSF zymography experiments were performed in triplicate. The mean and standard error of the data points were plotted against time, and analyzed using linear mixed model for significance after log or squared transformation.

## Results

### Doxycycline dose response

**Figure 1:**
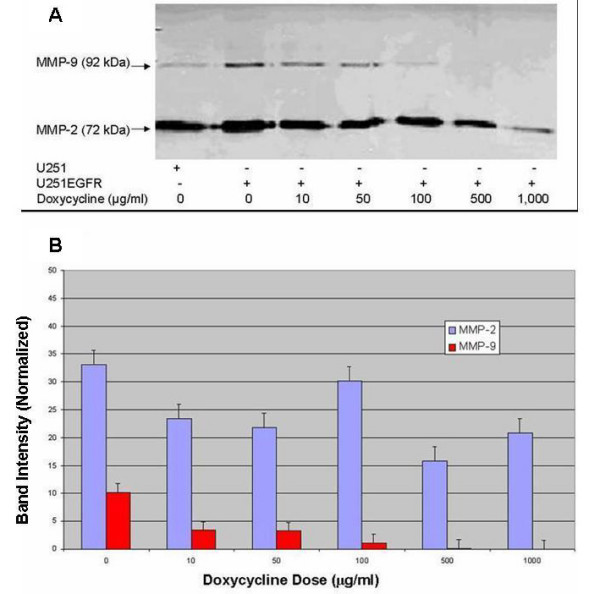
In vitro MMP inhibitory activity of doxycycline. (A) Zymography gel (light-dark reversed for clarity) of the *in vitro *effect on MMP-9 and MMP-2 activities by doxycycline. In U251 glioma cells overexpressing EGFR, doxycycline blocked completely MMP-9 gelanolytic activity at 500 μg/ml, while it only blocked partially MMP-2 activity at 1,000 μg/ml. (B) Normalized band intensity of gelanolytic activity of MMPs from U251 cells overexpressing EGFR at increasing concentrations of doxycyline. The IC_50 _was estimated to be 75 μg/ml for MMP-9 (red), while it was 500 μg/ml for MMP-2 (blue). Data are means +/- SEM, n = 4.

Doxycycline was studied *in vitro *using EGFR-transfected U251 glioma cells that secrete MMPs in order to evaluate the extent of MMP blockade. At a concentration of 100 μg/ml, doxycycline blocked > 50% MMP-9 gelanolytic activity from U251 cells overexpressing EGFR and completely abolished it at 500 μg/ml (Figure 1A &1B). The inhibitory activity of doxycycline for MMP-2 was less robust. There was at least 30% inhibition starting at 10 μg/ml but no complete block was observed at 1,000 μg/ml (Figures 1A &1B).

### Clinical trial result

Four patients completed 4, 3, 1, and 2 cycles of irinotecan, thalidomide, and doxycycline, respectively. The histologies consisted of 1 glioblastoma multiforme, 1 anaplastic astrocytoma, and 2 anaplastic oligodendrogliomas. All four patients tolerated treatment without a need for dose modification. Two patients died before tumor progression: one had CT angiogram-confirmed pulmonary thromboembolism, and another died during sleep, probably from pulmonary thromboembolism although no autopsy was granted. The other two did not develop thromboembolism. Their time to tumor progression was 4 and 6 months, while the survival of the cohort was 5, 8, 10, and 17+ months (Table 1). All were able to undergo neuroimaging evaluations and three lumbar punctures.

**Table 1:** Treatment data on patients treated with irinotecan, thalidomide, and doxycycline

**Patient**	**Age**	**Histology**	**Number of Cycles**	**TTP^a^**	**OS^a^**
1	61	anaplastic oligodendroglioma	4	6 months	17+ months
2	67	glioblastoma multiforme	3	4 months	10 months
3	54	anaplastic astrocytoma	1	N/A	5 month
4	34	anaplastic oligodendroglioma	2	N/A	8 months

^a^TTP = time to tumor progression; OS = overall survival

### Tumor response and perfusion by MRI

During the initial 12-week period, no detectable tumor progression was observed on gadolinium-enhanced T1-weighted MRI. The perfusion results from ASL also did not demonstrate evidence of tumor progression. Later neuroimaging results in Patients 1 and 2, at week 30 and 18 respectively, were consistent with tumor progression.

### CSF analysis

**Figure 2:**
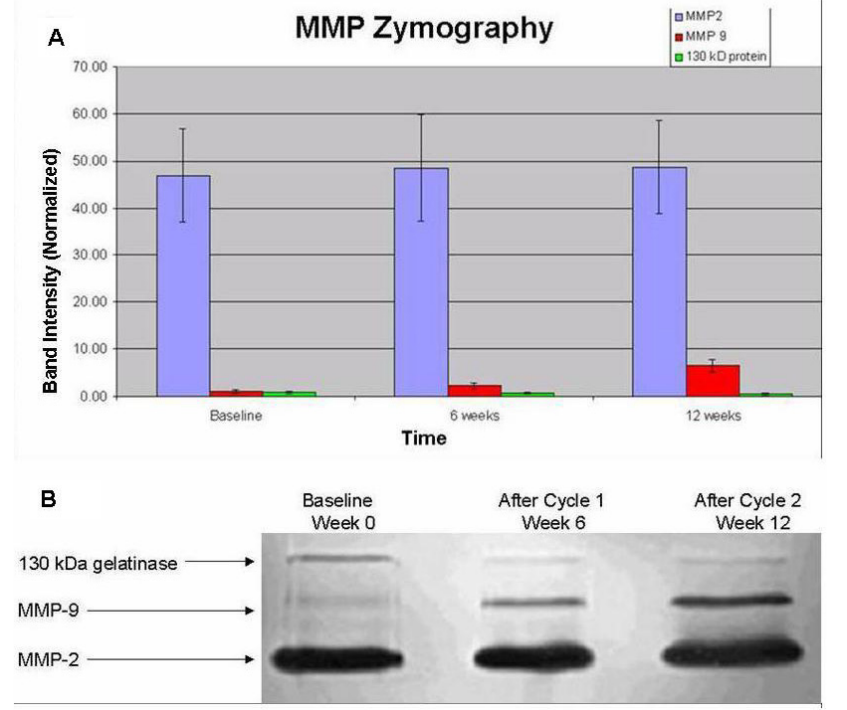
(A) Normalized band intensity of gelanolytic activity for CSF levels of MMP-2 (blue), MMP-9 (red), and 130 kDa MMP (green), and at baseline, after 6 weeks (1 cycle), and after 12 weeks (2 cycles) of treatment. Data are means +/- SEM, n = 4. (B) Representative zymography gel (light-dark reversed) of the CSF from Patient 1 showing stable gelanolytic activities from MMP-2 and 130 kDa MMP. But MMP-9 activity increased progressively over time.

All had negative CSF cytology for malignant cells. No activated MMP-2 (68 kDa) or activated MMP-9 (84 kDa) bands were detected on CSF zymography. The intensity of non-cleaved MMP-2 (72 kDa) and 130 kDa metalloproteinase gelanolytic bands, as measured by computerized densitometry, was stable at baseline, after 6 weeks (1 cycle), and after 12 weeks (2 cycles) of treatment (Figures 2A and 2B). There were no significant differences over time in MMP-2 and 130 kDa metalloproteinase activities. In contrast, there was a significant increase in MMP-9 (92 kDa) levels after 6 and 12 weeks of therapy compared to baseline (Figures 2A and 2B; *p *= 0.007). MMP-9 activity at week 12 was significantly higher than activities at baseline (*p *= 0.003) and at 6 weeks (*p *= 0.03), but there was no significant difference in MMP-9 activities between baseline and 6 weeks. However, when time was incorporated as a continuous variable in our analysis, there was an increasing trend in MMP-9 activities over time (*p *= 0.001).

## Discussion

Doxycycline, like tetracycline and its congeners, is a water-soluble non-specific competitive inhibitor of MMPs. Although doxycycline may dissociate from MMPs during SDS-PAGE, both MMP-2 and MMP-9 gelanolytic activities decreased as shown in our *in vitro *assay. To explain this phenomenon, one possibility is that doxycyline's small molecular weight and negative charge enable it to co-migrate with MMPs and inhibit MMP-2 and MMP-9 activities. An alternative explanation is that doxycycline has a non-competitive inhibitory effect on MMPs. Nevertheless, the high value of doxycycline IC_50 _estimated for MMP-2 and MMP-9 suggests that it blocks MMPs inefficiently.

The CSF zymography showed increasing MMP-9 gelanolytic activity over time. One source of this MMP-9 could come from the choroid plexus [19], and choroid plexus secretion may provide a basal level of MMP-9 in the CSF. Another source of this MMP-9 may be from bone marrow-derived hematopoietic progenitor cells helping tumor angiogenesis [20,21]. Friedberg et al [22] noted that activated MMP-2 and MMP-9 were primarily elevated in patients with glioblastoma multiforme and anaplastic astrocytomas with concurrent positive CSF cytology for malignant cells. Similarly in lymphomatous meningitis, MMP-9, but not MMP-2 or 130 kDa MMP, correlated with disease activity [18]. In both studies, MMP-9 activity appears to be more sensitive than MMP-2 for malignancies in the CNS, as a basal level of MMP-2 gelanolytic activity was detected in healthy normal controls [18,22]. This is in contradiction to an immunohistochemical study of astrocytic brain tumors by Kunishio et al [23] who demonstrated that invasion correlated with MMP-2 but not MMP-9. But this difference may be the result of analysis performed on CSF samples versus brain tumor specimens. In our patients, no activated MMP-9 was detected and none of our patients had positive CSF cytology for malignant cells. Therefore, the increasing MMP-9 activity in our cohort may reflect malignant glioma disease activity. There was also a slight decrease in the 130 kDa gelatinase band over time but this was not significant when averaged among all four patients.

In our patients, it is significant to note that CSF MMP-9 activity began to rise after 6 weeks of treatment despite relatively stable gadolinium enhancement and ASL perfusion. Changes in gadolinium enhancement and ASL perfusion were detected much later in two patients. Perhaps increasing MMP-9 activity portends tumor progression and it could be used as a marker to detect early tumor progression. Additional CSF analysis beyond cycle 2 would be helpful to address this issue. Furthermore, because angiogenesis depends on an ensemble of cellular functions, including endothelial cell proliferation, adhesion and migration in the extracellular matrix, morphogenesis, and MMP secretion, doxycycline may help to potentiate the antiangiogenesis effect of thalidomide. But the optimal dose of doxycycline to block glioma invasion and angiogenesis remains to be determined.

In this clinical trial, patient enrollment had to be terminated due to side effects. After enrolling four patients, one developed progressive pulmonary thromboembolism despite anticoagulation with warfarin, while another probably died from the same adverse event during sleep. These two adverse events could be a result of the malignant gliomas since the incidence of thromboembolism is 24% in these patients [24]. Furthermore, both patients were on concurrent dexamethasone and the risk of thromboembolism from taking thalidomide and dexamethasone was estimated to be up to 30% in patients with multiple myeloma [25,26]. Although most reports came from patients with multiple myeloma, thalidomide- and dexamethasone-induced thromboembolism may not be unique to this population as patients with mantle cell lymphoma [27] and Waldenström's macroglobulinemia [28] also encountered an increased rate of thromboembolism. However, low molecular weight heparin may lower this risk. When patients with multiple myeloma were treated with thalidomide, chemotherapy, and nadroparine, their incidence of thromboembolism decreased to 10% [29].

## Conclusion

In our cohort of four patients with recurrent malignant gliomas, CSF MMP-9 activity progressively increased before any evidence of tumor progression on MRI. This finding suggests that CSF MMP-9 could be a marker for early detection of tumor progression. The optimal dose of doxycycline necessary to block all MMP activities is unknown.

## Abbreviations

AIDS: Acquired immunodeficiency syndrome;

ASL: Arterial spin labeling;

DMEM: Dulbecco's modified Eagle's medium;

EGFR: Epidermal growth factor receptor;

FDA: Food and drug administration;

HIV: Human immunodeficiency virus;

IC_50: _50% inhibitory concentration;

KPS: Karnofsky performance status;

MMP: Matrix metalloproteinase;

MRI: Magnetic resonance imaging;

PAGE: Polyacrylamide gel electrophoresis;

SDS: Sodium dodecyl sulfate;

S.T.E.P.: System for thalidomide education and prescribing safety.

## Competing interests

The author(s) declare that they have no competing interests.

## Authors' contributions

ETW, DL, AT, and JKW actively participated in planning, design, conducting experiments and clinical trial, analyzing the data, and preparation of manuscript. DA performed analysis of the MRI and ASL perfusion images. LB, JB, and SG carried out clinical trial data collection, patient care, statistical support, and preparation of manuscript. All authors have read and approved the manuscript.
